# Core/Shell Structure of Ni/NiO Encapsulated in Carbon Nanosphere Coated with Few- and Multi-Layered Graphene: Synthesis, Mechanism and Application

**DOI:** 10.3390/polym8110381

**Published:** 2016-11-09

**Authors:** Ferial Ghaemi, Luqman Chuah Abdullah, Paridah Tahir

**Affiliations:** 1 Institute of Tropical Forestry and Forest Products (INTROP), Universiti Putra Malaysia (UPM), 43400 Serdang, Malaysia; luqmanchuah@gmail.com (L.C.A.); parida.introp@gmail.com (P.T.); 2 Department of Chemical and Environmental Engineering, Universiti Putra Malaysia (UPM), 43400 Serdang, Malaysia

**Keywords:** carbon nanosphere, graphene, core/shell structure, chemical vapor deposition, polypropylene

## Abstract

This paper focuses on the synthesis and mechanism of carbon nanospheres (CNS) coated with few- and multi-layered graphene (FLG, MLG). The graphitic carbon encapsulates the core/shell structure of the Ni/NiO nanoparticles via the chemical vapor deposition (CVD) method. The application of the resulting CNS and hybrids of CNS-FLG and CNS-MLG as reinforcement nanofillers in a polypropylene (PP) matrix were studied from the aspects of mechanical and thermal characteristics. In this research, to synthesize carbon nanostructures, nickel nitrate hexahydrate (Ni(NO_3_)_2_·6H_2_O) and acetylene (C_2_H_2_) were used as the catalyst source and carbon source, respectively. Besides, the morphology, structure and graphitization of the resulting carbon nanostructures were investigated. On the other hand, the mechanisms of CNS growth and the synthesis of graphene sheets on the CNS surface were studied. Finally, the mechanical and thermal properties of the CNS/PP, CNS-FLG/PP, and CNS-MLG/PP composites were analyzed by applying tensile test and thermogravimetric analysis (TGA), respectively.

## 1. Introduction

The discovery of new carbon forms has triggered a great interest in the carbon material science community [1,2]. Among these nanomaterials, carbon nanospheres (CNS) and graphene (G) have drawn great attention due to their potential applications in different fields of science such as lithium-ion batteries, supercapacitors, polymer composites, medical sciences, etc., which result from their excellent properties such as superior chemical stability, thermal insulation, low density, as well as high compressive strength [3,4,5,6,7,8].

Among the methods employed to synthesize CNS and G, such as the arc plasma technique [9,10], hydrothermal reaction [11,12], spray pyrolysis [13,14] and chemical vapor deposition (CVD) [15,16,17,18,19,20], CVD as a simpler, lower-cost and easier-to-implement method shows some advantages including uniform surface coating by nanomaterials and homogenous growth of material with high purity and large yield [21,22]. In the CVD technique, certain catalysts are used for nanomaterial fabrication including Fe, Ni and Co for growing CNS, and Ni, Co and Cu for growing graphene [17,18].

Growing graphene as a two-dimensional structure on other nanomaterials including carbon nanospheres, carbon nanofibers and carbon nanotubes makes hybrid nanomaterials with remarkable properties, such as thermal and mechanical properties, which have the potential to be used in many fields of science and engineering [23,24,25,26].

On the other hand, based on the number of graphene layers, graphene sheets have different types including single-layered (one layer), double-layered (two layers), few-layered (three to five layers) and multi-layered (six to 10 layers), each of which have different properties [27,28,29]. So, in the CVD technique, using certain parameters such as temperature, different shapes of carbon nanomaterials including CNS and G are obtained. Also, by altering different reaction times, different layers of graphene are achieved.

To date and to the best of the authors’ knowledge, there is no study reporting the synthesis of carbon nanosphere-few layer graphene (CNS-FLG) and carbon nanosphere-multi layer graphene (CNS-MLG) hybrids using the CVD method and studying its growth mechanisms. In some existing research, authors have mixed CNS and G sheets to achieve CNS-G, but in this research, the CVD technique was employed to synthesize CNS, followed by few-layered and multi-layered graphene on its surface to produce CNS-FLG and CNS-MLG. Besides, the mechanisms to synthesize few- and multi-layered graphene on the grown CNS were studied. Finally, the nanomaterials were used as nanofillers in a polymer matrix to produce a nanocomposite with improved mechanical properties and thermal stability [30,31]. Furthermore, the resulting nanomaterial hybrids were used as reinforcement nanofillers in a polypropylene matrix to improve the polymer’s properties.

In order to characterize the produced nanomaterial by its surface morphology, structure, components, number of graphene layers, size and graphitization, scanning electron microscopy (SEM), energy dispersive X-ray (EDX), transmission electron microscopy (TEM) and Raman spectroscopy were used.

## 2. Materials and Methods

The CVD reactor (Downwell Resources Sdn. Bhd, Puchong, Malaysia) was used to synthesize carbon nanomaterials including carbon nanosphere, and few layered and multi layered graphene. In order to obtain different morphologies and sizes of the nanomaterials, CVD parameters such as reaction temperature and time should be changed. Therefore in this case, the temperature was set to range from 950 to 1050 °C to synthesize CNS and G, respectively. Besides, the number of graphene layers was changed from few layers to multi layers by changing the time from 30 to 50 min.

Initially, quartz substrate (the boat of the CVD reactor) was immersed in Ni(NO_3_)_2_·6H_2_O solution for 2 h. Then it was dried at 100 °C under airflow for 1 h. After that, the boat was placed in the middle of the CVD reactor and the system was set. Then, the furnace was turned on to increase the temperature and at this time, N_2_ gas was released into the CVD chamber to remove O_2_ inside the reactor. When the temperature reached about 300 °C, the surface of the boat was calcinated and the nitrate compound was removed and the Ni/NiO particles remained. When the temperature reached 950 °C, the CNS fabrication started for 30 min by the use of acetylene (carbon source), with a flow rate of 50 standard cubic centimeters per minute (sccm) under flowing H_2_/N_2_ (flow rates of 50, 100 sccm) at atmospheric pressure (1 bar). After that, at the end of reaction time, the acetylene flow was stopped and the temperature was increased to 1050 °C under flowing C_2_H_2_/H_2_/N_2_ (flow rates of 50, 50, 100 sccm) to synthesize graphene sheets. To synthesize few layered and multi layered graphene, different time durations (30 to 50 min) were used. In order to analyze the structural characteristics and graphitization of the resultant CNS, CNS-FLG, CNS-MLG, Raman spectroscopy (alpha 300 R, WITec, Ulm, Germany) was used. Raman spectrometer with a green Argon laser excitation source having a wavelength of 532 nm to observe the spectrum of the unstressed specimen.

Besides, the morphology and components of the product were inspected using scanning electron microscope (SEM) (FEI Company, Hillsboro, OR, USA), energy dispersive X-ray (EDX) (Oxford INCA EDX 300, Singapore)and transmission electron microscope (TEM) (HITACHI Limited, Tokyo, Japan).

The last part of the experimental work was to prepare the polymer composite. In this section, Thermo Haake Poly Drive R600/610 (LabX, Midland, ON, Canada) as mixer was used to melt and blend the polypropylene (PP) at 180 °C with a 55 rpm rotor speed for 5 min. After that, the nanofillers (5 wt %) were added into the mixer and was blended again for 15 min [32]. Then, the mixture was put inside a mold of 15 cm × 15 cm area and 1 mm thickness and melted again at 180 °C under 150 kg·cm^−2^ pressure by the use of a HSINCHU hot press machine (Tradekey, HsinChu, Taiwan) and then cooled. Finally, based on the guideline in ASTM D638 standard, the specimen and cut into dog bone shape [33].

In order to perform mechanical tests, an Instron Universal Testing Machine (Instron, Canton, MA, USA) was used with a crosshead speed of 5 mm/min to determine and measure the strength modulus and tensile stress of the PP, CNS/PP, CNS-FLG/PP and CNS-MLG/PP composites [34]. Furthermore, to determine the thermal resistance of the produced nanocomposites, thermal gravimetric analysis (TGA) was employed using a Mettle Stare SW 9.10 thermal gravimetric analyzer (Polaris Parkway, Columbus, OH, USA) [35]. Firstly, to remove humidity from the composite, it was heated to 200 °C for few minutes. After that, a heating program was applied from 25 to 1000 °C with a heating rate of about 10 °C/min under nitrogen flow.

## 3. Results and Discussion

### 3.1. Nanomaterial Characterization

**Figure 1:**
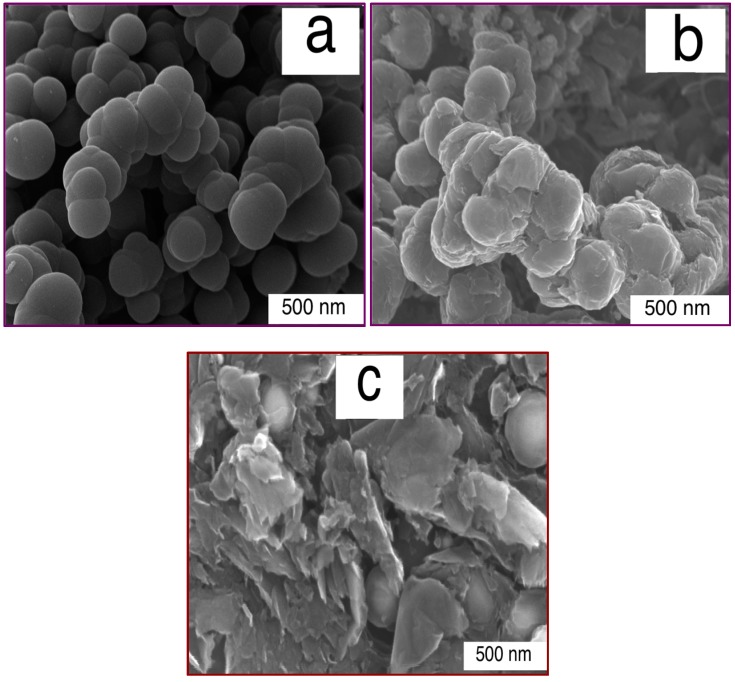
SEM images of (**a**) CNS (carbon nanospheres); (**b**) CNS-FLG (Carbon nanosphere-few layer graphene) and (**c**) CNS-MLG (Carbon nanosphere-multi layer graphene).

**Figure 2:**
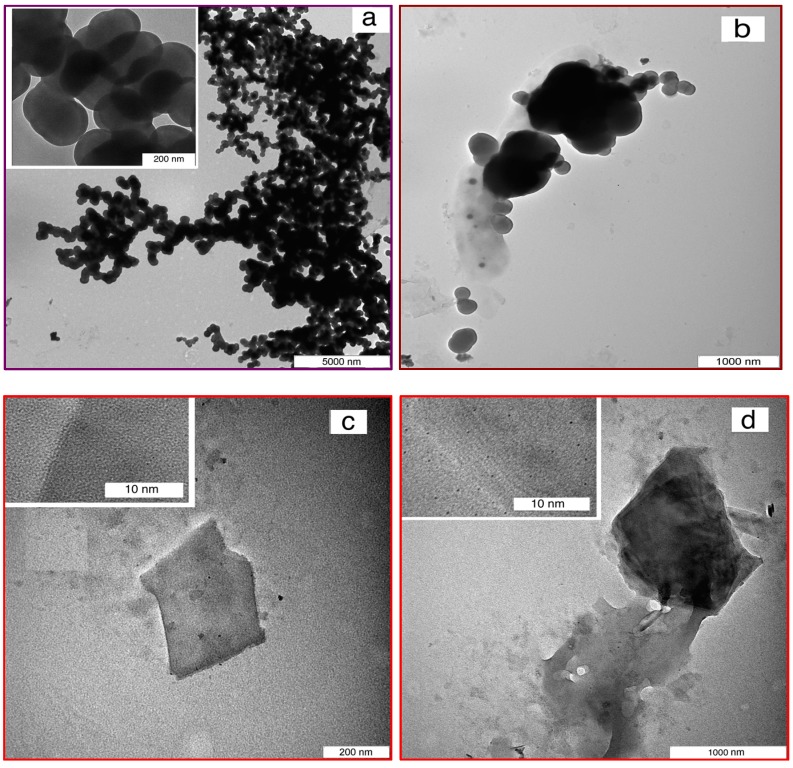
TEM images of (**a**) CNS; (**b**) CNS coated with MLG; (**c**) FLG and (**d**) MLG.

The agglomeration of produced CNS coated with the grown few- and multi-layered G formed by CVD was demonstrated by electron microscopy (SEM and TEM) images. Figure 1a–c show the SEM images of the CNS, CNS-FLG and CNS-MLG, and Figure 2a–d illustrate the TEM images of the CNS, CNS-coated with graphene sheets, FLG and MLG, respectively.

The synthesized CNS had a spherical shape, which made it a porous structure (Figure 1a). Besides, the synthesis of graphene sheets on the CNS surface resulted in a rough structure on the CNS surface. Therefore, by growing few-layered graphene on the CNS surface, a thin layer of graphene sheet covered the surface of the CNS (Figure 1b), while the growth of multi-layered graphene led to thick layers on the CNS surface, as well as graphene sheets occupying the space between CNS (Figure 1c and Figure 2b). This claim was proven by the width of the graphene sheet in Figure 2c,d, whereby the width and number of graphene layers of the multi-layered graphene were greater than those of the few-layered graphene. So, the diameter and size of the produced nanomaterials were approximately 200 nm in diameter for CNS (Figure 2a), and 300 nm × 400 nm and 1000 nm × 2000 nm for the FLG sheet (Figure 2c) and MLG sheet (Figure 2d), respectively.

**Figure 3:**
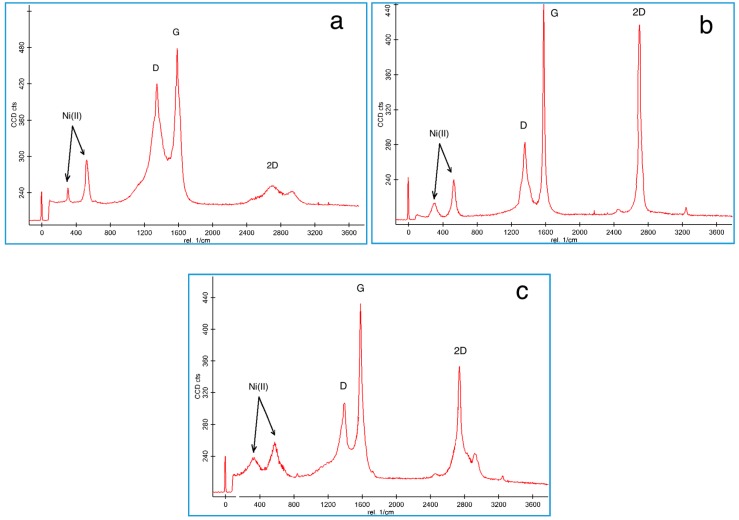
Raman spectra of (**a**) Ni/CNS; (**b**) Ni/CNS-FLG, and (**c**) Ni/CNS-MLG.

Raman spectroscopy is a powerful yet relatively simple method to characterize the thickness and crystalline quality of graphene layers [19]. Figure 3 depicts the Raman spectra for characterizing the different nanomaterials including CNS, FLG-CNS and MLG-CNS with embedded Ni/NiO nanoparticles as the catalyst. The spectra for Ni (II) particles which appeared at 317 and 555 cm^−1^ corresponded to the E_g_ and A_1g_ modes, respectively [36], while the peaks at 1350, 1580 and 2650 cm^−1^ corresponded to the D peak (breathing modes of the sp^2^ atom due to defective structure), G peak (E_2g_ stretching mode of the graphitic crystalline structure) and 2D peak (Raman signature of graphitic sp^2^ materials) of the carbonic nanomaterials, respectively [37].

In order to estimate the degree of graphitization, the sp^2^ cluster size and number of graphene layers of the carbonic structures, the intensity ratio of the D peak and G peak (*I*_D_/*I*_G_) and the intensity ratio of the 2D peak and G peak (*I*_2D_/*I*_G_) were calculated [19,38]. It is observed that when I_D_/I_G_ decreased, the graphitization increased. As well, by increasing the *I*_2D_/*I*_G_ ratio, the number of the graphene layers decreased.

The Raman spectra obtained for CNS and CNS coated with few-layered and multi-layered G sheets are revealed in Figure 3a–c, respectively. Based on the Raman spectra, the *I*_D_/*I*_G_ ratio was about 0.87 and the *I*_2D_/*I*_G_ ratio was 0.52 for CNS, the *I*_D_/*I*_G_ ratio was about 0.63 and the *I*_2D_/*I*_G_ ratio was 0.94 for CNS-FLG, and the *I*_D_/*I*_G_ ratio was about 0.72 and the *I*_2D_/*I*_G_ ratio was 0.81 for CNS-MLG. Therefore, it can be concluded that the few growing layers of graphene sheets on the carbon nanosphere surface led to the increment of the graphitization of the hybrid.

**Figure 4:**
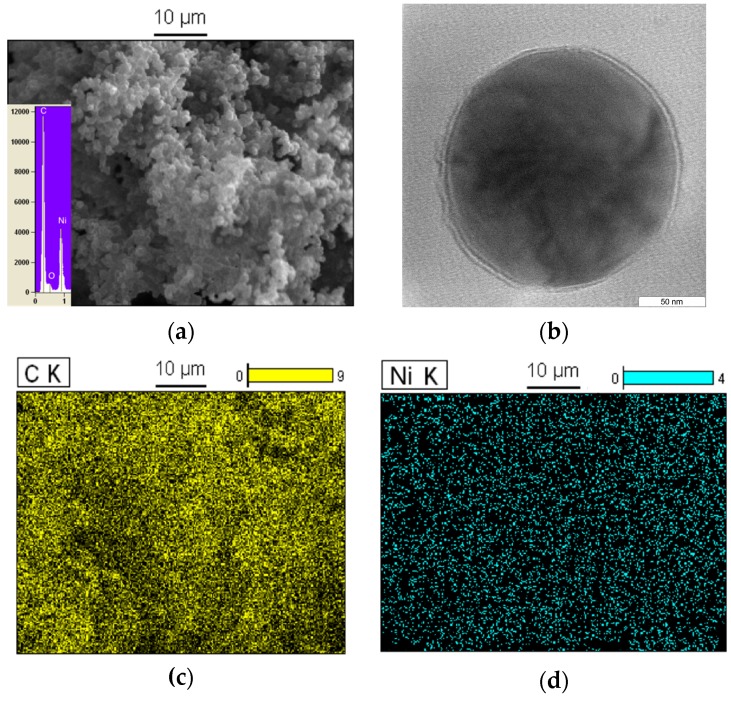
SEM/EDX image of Ni/C nanospheres (**a**); TEM image of Ni/C nanospheres (**b**); elemental mapping images of C at 950 °C (**c**) and Ni at 950 °C (**d**).

The SEM/EDX image of the carbon nanospheres at 950 °C under C_2_H_2_/H_2_/N_2_ flow is revealed in Figure 4a, which shows that these carbon nanospheres were linked to each other. EDX spectroscopy indicates that the carbon nanosphere was composed of C, Ni and O atoms with about a 69:25:6 atomic ratio and the C element was most predominant. Besides, the TEM image in Figure 4b shows that carbon nanospheres with a diameter of about 200 nm surrounded the Ni and NiO particles (black part). Moreover, elemental mapping images based on carbon nanospheres proved the distribution of the Ni and C atoms (Figure 4c,d). These results indicate that Ni elements existed in the form of nanoparticles with about 25% Ni particle content.

**Figure 5:**
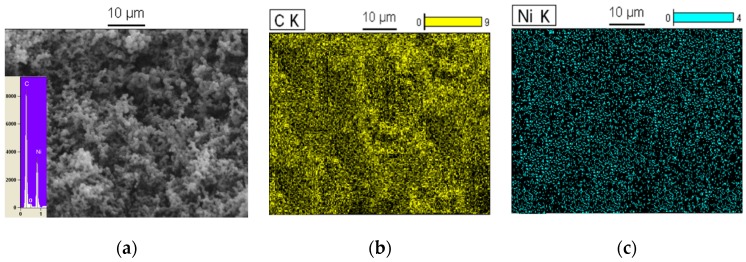
SEM/EDX image of Ni/C nanospheres (**a**); elemental mapping images of C at 1050 °C (**b**) and Ni at 1050 °C (**c**).

On the other hand, based on the SEM/EDX images in Figure 5a, carbon nanospheres at 1050 °C under H_2_/N_2_ flow and without C_2_H_2_ flow were composed of C, Ni and O with an atomic ratio of approximately 66:30:4. This result states that the amount of Ni particles increased while the O atoms decreased in comparison with carbon nanospheres at 950 °C. Hence, by increasing the temperature to 1050 °C under H_2_/N_2_ flow, more Ni salt was converted to NiO and more NiO was converted into Ni particles. EDX spectroscopy and elemental imaging in Figure 5b,c prove this claim. So, the percentage of Ni nanoparticles in the carbon nanospheres was 30%, and it increased by about 5% in comparison with the previous carbon nanospheres, which had 25% Ni content, and which revealed the presence of Ni particles around and on top of the carbon nanosphere surface.

After that, by inserting the C_2_H_2_ flow into the CVD reactor, the graphene sheets were grown on the Ni particles placed around and on top of the CNS surface.

### 3.2. Mechanism

**Figure 6:**
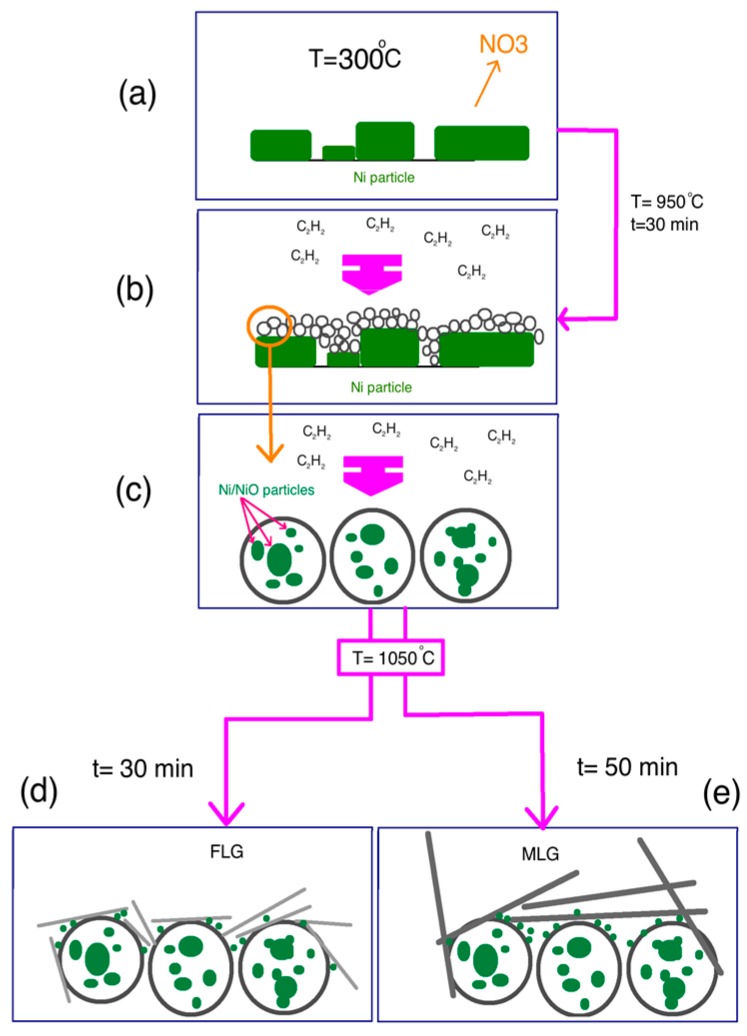
Schematic illustration of process: (**a**) NO_3_ elimination and formation of Ni/NiO, (**b**) synthesis of carbon nanosphere, (**c**) zoon in of carbon nanosphere encapsulated Ni/NiO particles, (**d**) few-layer graphene and (**e**) Multi-layer graphene productions on CNS.

According to the results for characterization, the schematics for the formation of the different carbon nanomaterials including CNS, CNS-FLG and CNS-MLG are depicted in Figure 6. As a matter of fact, the defect structure of graphene has a negative effect on the mechanical and thermal properties of the composite; hence, the large amount of the crystal part of graphene leads to significantly improved mechanical and thermal properties. Based on the Raman results, the defect structures are present in a lower amount in comparison with graphitization (compare the height of the D peak and the G peak).

Figure 6a shows the substrate coated with Ni(NO_3_)_2_·6H_2_O particles. When the temperature is increased to about 300 °C and the H_2_/N_2_ gases are inserted into the CVD reactor, the decomposing process of Ni(NO_3_)_2_·6H_2_O particles starts as follows [39].

Water separation:

Ni(NO_3_)_2_·6H_2_O = Ni(NO_3_)_2_·2H_2_O + 4H_2_O

Decomposition:

Ni(NO_3_)_2_·2H_2_O = Ni(NO_3_)(OH)_1.5_·O_0.25_·H_2_O+ 0.25H_2_O + NO_2_

Ni(NO_3_)(OH)_1.5_·O_0.25_·H_2_O = 0.5Ni_2_O_3_ + HNO_3_ + 1.25H_2_O

Oxide decomposition to NiO:

3Ni_2_O_3_ = 2Ni_3_O_4_ + 0.5O_2_

Ni_3_O_4_ = 3NiO + 0.5O_2_

Reduction with H_2_/N_2_ to Ni:

Ni_3_O_4_ = 3NiO + 0.5O_2_

3NiO + 3/2H_2_ = 3Ni + 3/2H_2_O

After that, at a temperature of 950 °C, acetylene gas was released into the CVD reactor and the H–C–C–H bonds in the C_2_H_2_ molecules started to break up to form C atoms (Figure 6b). During the reaction time, C atoms collected together to form a nanosphere of the deposited sp^2^ structure on active Ni atoms, and then surrounded the Ni/NiO nanoparticles (Figure 6c). Finally, CNS with a network structure were generated, in which Ni/NiO nanoparticles were embedded in the carbon nanosphere matrix. After that, the acetylene flow was stopped. Then, to grow the graphene sheets on the CNS surface, the temperature was increased to about 1050 °C. While the reactor was reaching this temperature, more Ni particles were converted into Ni/NiO nanoparticles and deposited on the produced carbon nanosphere surface. Finally, when the temperature reached 1050 °C, acetylene gas filled the chamber and the graphene sheets started to grow on the CNS surface for 30 min and 50 min to form few-layered graphene and multi-layered graphene, respectively (Figure 6d,e) [18].

### 3.3. Composite Characterization

**Figure 7:**
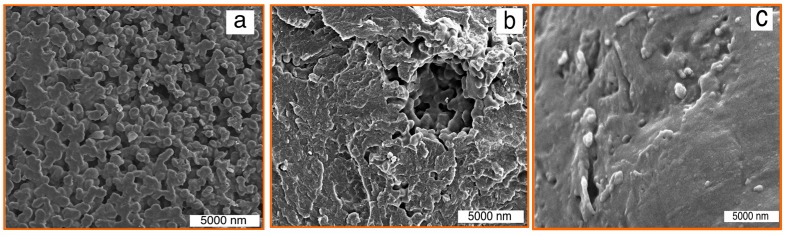
SEM pictures of different composites: (**a**) CNS/PP; (**b**) CNS-FLG/PP and (**c**) CNS-MLG/PP.

SEM images of polypropylene (PP) composites including CNS/PP, CNS-FLG/PP and CNS-MLG/PP are depicted in Figure 7. In these images, the dispersion of nanofillers in the PP matrix and the interfacial adhesion between the nanofillers and polymer matrix were observed. According to Figure 7a, the synthesized CNS had a good interfacial interaction with PP; however, it led to a porous polymer matrix structure. Besides that, the graphene sheets on the CNS surface enhanced the interaction with PP. In fact, the presence of the graphene on the CNS makes a rough surface in comparison with the smooth surface of neat CNS, which leads an increased surface area. Besides, the interaction of graphene-CNS with polypropylene with the porous structure of CNS and the hard structure of graphene leads to a significantly enhanced interaction between the polypropylene and nanofillers.

Therefore, regarding Figure 7b, the rough surface of the few-layered graphene sheets on the CNS surface led to a greater surface area, which proves the high interfacial adhesion between the nanofiller and polymer matrix. Moreover, the influence of using multi-layered graphene on the CNS surface as a nanofiller on the interaction with the polymer matrix is shown in Figure 7c.

Indeed, the multi-layered graphene covered the agglomerate of CNS (refer Figure 1c) and also occupied the spaces between CNS. Therefore, when this nanofiller mixed with polypropylene, the porosity of the nanocomposite significantly decreased in comparison with the other mentioned nanocomposites. Therefore, the presence of graphene sheets as nanofillers and as an interlocking factor with the polymer matrix is important, but not as much as the number of graphene layers.

**Figure 8:**
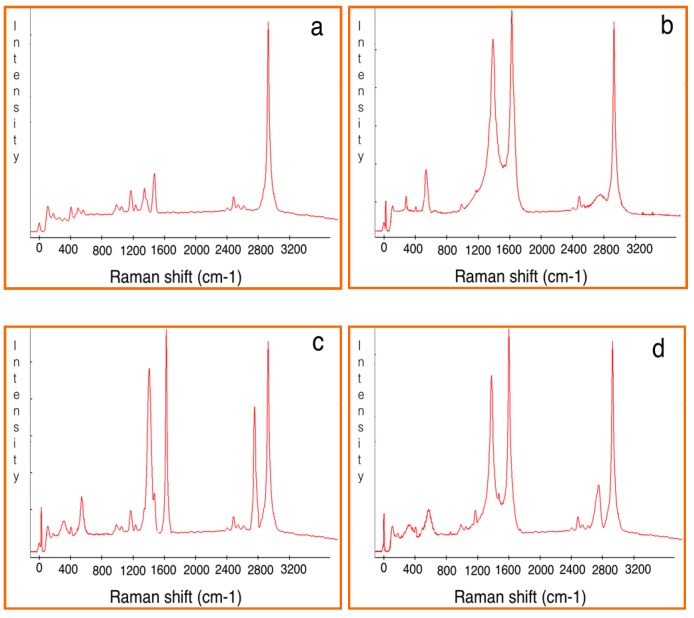
Raman spectroscopy of (**a**) PP; (**b**) CNS/PP; (**c**) CNS-FLG/PP and (**d**) CNS-MLG/PP.

Moreover, Raman spectroscopy was employed to further analyze the interaction between the different nanofillers and polymer matrix, which should be reflected in the change in peak shift or peak width [23]. Based on the results in Figure 8a–d, the peaks related to nanocomposites were sharper than those of pure polymer because of the resonance and absorbance effects of the nanofillers. In other words, the peaks of the Raman spectra for the nanocomposite were significant because of the high intensity of the nanofillers with the polymer matrix, while the peak for pure polymer did not appear. Subsequently, the existence of nanofillers in the polymer matrix led to an increment in the intensity of the G and 2D peaks in the polymer composite because of the enhanced interaction between the polymer and the filler. Furthermore, the presence of graphene sheets on CNS improved the interaction with the polymer matrix, and also it was finally understood that CNS-FLG had a stronger adhesion to the polymer matrix in comparison to CNS-MLG.

**Figure 9:**
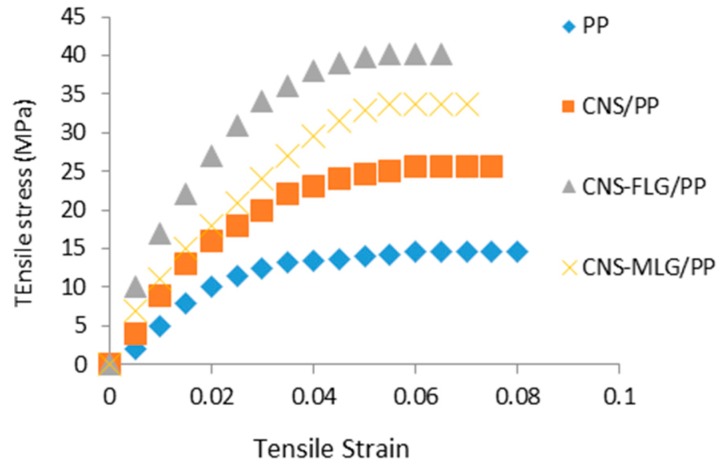
Tensile stress–strain graph of PP, CNS/PP, CNS-FLG/PP and CNS-MLG/PP.

Based on the results achieved from the mechanical test, it was found that the nanofillers improved the mechanical properties (see Table 1 and Figure 9). Besides, the study on the stiffness of the composite fabricated from the different nanofillers, including CNS, CNS-FLG and CNS-MLG, revealed that CNS coated with graphene layers led to a significant improvement in the tensile test results in comparison with neat CNS because of the high interfacial adhesion. Also, the strength of CNS-FLG/PP and CNS-MLG/PP nanocomposites was greater than that of the CNS/PP nanocomposite due to the presence of graphene layers on the CNS surface, which led to a high stress transfer between the CNS and polymer. On the other hand, the few-layered graphene, which had a higher surface area in comparison with multi-layered grapheme, had a stronger interaction with PP, leading to the CNS-FLG/PP nanocomposite having the highest tensile strength and stress [18]. Note that the tensile strength of a material is the maximum amount of tensile stress that it can take before breaking, and the Young’s modulus is the ratio of stress upon strain.

**Figure 10:**
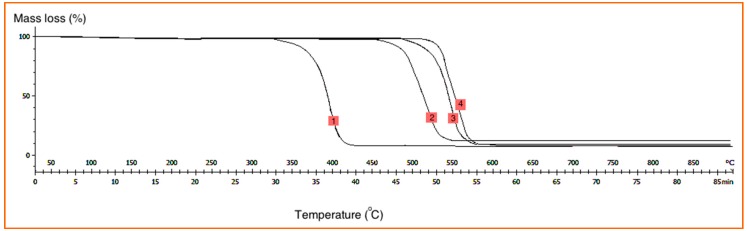
TGA graphs of the various polymer composites (1: PP; 2: CNS/PP; 3: CNS-MLG/PP and 4: CNS-FLG/PP); Horizontal axis: Temperature (°C); Vertical axis: Loss weight percentage (wt %).

According to the thermogravimetric analysis (TGA) results, it was found that the existence of nanofillers in the polymer matrix led to improved thermal resistance of the polymer nanocomposite due to the high heat absorption capacity of the carbon nanomaterials (see Figure 10). Once the specimen absorbs a certain amount of heat, thermal degradation happens, leading to a single degradation step. Then, the structure of the polymer composite decomposed. Based on Figure 10, the neat PP was broken down at about 330 °C and degraded completely at 430 °C without any char left. The weight loss of the CNS/PP, CNS-MLG/PP and CNS-FLG/PP composites started at 450, 500 and 530 °C, and completed at 550, 580 and 590 °C, respectively. Based on the results, CNS-FLG/PP had greater thermal resistance than CNS/PP and CNS-MLG/PP composites because of the high thermal resistance of the few-layered graphene in comparison to other nanofillers. Therefore, by growing few-layered graphene on CNS, the thermal resistance of the nanocomposite was significantly improved.

## 4. Conclusions

The most important contribution of this research is the synthesis of the few- and multi-layered graphene on the CNS, as well as the synthesis of the CNS encapsulating the Ni/NiO particles using the chemical vapor deposition technique. The reaction commenced by depositing carbon atoms on the Ni particle surface. Also, CNS was grown at 950 °C for 30 min and FLG and MLG were synthesized on CNS surfaces at 1050 °C for 30 and 50 min, respectively. Moreover, we studied the mechanism for hybrid growth and developed a model for growing a carbon nanosphere–graphene hybrid, which encapsulated the Ni nanoparticles. Regarding the results, firstly, CNS was grown on Ni/NiO nanoparticles at 950 °C. Thereafter, by increasing the temperature under H_2_/N_2_ gas flow, more Ni/NiO evaporated around the produced CNS, which led to the synthesis of graphene sheets on the CNS surface. Finally, the resultant nanomaterials including CNS, CNS-FLG and CNS-MLG were used as reinforcing nanofillers in the polymer matrix to improve the mechanical and thermal properties of the polypropylene matrix. According to the obtained outputs, it is concluded that CNS-FLG had more a significant role in improving the properties of the polymer composite such as tensile strength, strain and thermal resistance.

## Figures and Tables

**Table 1:** Mechanical results of the different nanocomposites (5% nanofiller, 95% PP).

Sample	Tensile stress (MPa)	Young’s modulus (MPa)
PP	15.1 ± 0.3	480.2 ± 11.7
CNS/PP	25.6 ± 0.4	830.2 ± 31.2
CNS-FLG/PP	73.8 ± 0.6	1,243.6 ± 57.1
CNS-MLG/PP	40.1 ± 0.7	1,097.5 ± 48.2
